# The effect of 7-ketocholesterol and 25-hydroxycholesterol on the integrity of the human aortic endothelial and intestinal epithelial barriers

**DOI:** 10.1007/s00011-013-0660-x

**Published:** 2013-09-28

**Authors:** Maciej Chalubinski, Katarzyna Zemanek, Wojciech Skowron, Katarzyna Wojdan, Paulina Gorzelak, Marlena Broncel

**Affiliations:** Department of Internal Diseases and Clinical Pharmacology, Medical University of Lodz, Ul. Kniaziewicza 1/5, 91-347 Lodz, Poland

**Keywords:** Oxidized cholesterols, Endothelium, Intestinal epithelium, Barrier functions, Impedance, Apoptosis

## Abstract

**Objective and design:**

The damage of barrtier tissues, such as the vascular endothelium and intestinal epithelium, may lead to disturbances of local immune homeostasis. The aim of the study was to assess and compare the effect of oxidized cholesterols (7-ketocholesterol and 25-hydroxycholesterol) on the barrier properties of human primary aortic endothelium (HAEC) and intestinal epithelium Caco-2 cells using a real-time cell electric impedance sensing system (RTCA-DP).

**Materials and methods:**

HAEC and Caco-2 cells were stimulated with 7-ketocholesterol and 25-hydroxycholesterol by the RTCA-DP system. Apoptosis was assessed by flow cytometry and cell monolayer morphology was assessed under a light microscope.

**Results:**

7-ketocholesterol decreased impedance (nCI) in both the endothelium and epithelium. However, the decrease was more profound in the endothelium. Similarly, although 25-hydroxycholesterol decreased nCI in both the endothelium and epithelium, the effect was weaker than that of 7-ketocholesterol, which caused extensive damage to the endothelial monolayer, while 25-hydroxycholesterol caused partial damage and did not affect the epithelial monolayer. 7-ketocholesterol, but not 25-hydroxycholesterol, increased endothelial cell apoptosis and decreased the viability of endothelial cells. However, 7-ketocholesterol and 25-hydroxycholesterol decreased epithelial cell apoptosis and increased viability.

**Conclusion:**

Oxidized cholesterols destroy the HAEC, but not the Caco-2 epithelial barrier, via cell apoptosis dependent on the site of oxidation. Damage to the endothelium by oxidized cholesterol may disrupt local homeostasis and provide open access to inner parts of the vascular wall for lipids, other peripheral blood-derived agents, and immune cells, leading to inflammation and atherogenesis.

## Introduction

Decreased integrity and increased permeability of barrier tissues such as the endothelium and epithelium may lead to serious disturbances of local homeostasis, causing various pathologies, including inflammation. The intestinal epithelium interphase has been observed to be disintegrated by inflammatory bowel disease (IBD), food allergy, and bacterial infections [1, 2]. Similarly, disturbances of the barrier functions of the vascular endothelium have been evidenced as important components of multiple sclerosis and diabetic retinopathy [3, 4].

Both epithelial and endothelial barriers are exposed to oxysterols—products of cholesterol oxidation playing a crucial role in atherosclerosis development. Of them, the ones most commonly found in food are 7-oxygenated sterols, such as 7-ketocholesterol, 7α-hydroxycholesterol and 7β-hydroxycholesterol [5]. Other oxygenated sterols such as 25-hydroxycholesterol, are present in smaller amounts. Upon absorption by the intestinal epithelium, they reach the plasma, where they are then transported by lipoproteins affecting the vascular endothelium, and finally are distributed to tissues [6].

Both 7-ketocholesterol and 25-hydroxycholesterol have been demonstrated to possess pro-inflammatory properties, as they induce IL-1β and IL-8 synthesis by different cell types [7, 8]. However, some differences exist in their potency; for example, while 7-ketocholesterol induces apoptosis of various cell types, 25-hydroxycholesterol has been shown to demonstrate less pronounced cytotoxicity dependent on cell type [7, 9, 10]. 25-hydroxycholesterol can be a more potent agent and inductor of IL-8 release [7, 10]. Therefore, both 7-ketocholesterol and 25-hydroxycholesterol may trigger the immune inflammatory processes within the subepithelial and subendothelial spaces, leading to, respectively, intestinal and vascular wall inflammation. However, the influence of 7- and 25-oxigenated cholesterols on endothelial and epithelial barrier functions and its significance has not been widely elucidated.

The aim of our study was to assess and compare the effect of two different oxysterols, 7-ketocholesterol and 25-hydroxycholesterol, on the properties of both intestinal epithelial Caco-2 cells and the primary human aortic endothelial barrier. To do so, a novel cell-based method was used to enable permanent monitoring of the condition and integrity of the adherent cell monolayer: real-time cell electric impedance sensing system ×CELLigence (RTCA-DP). The second aim of the study was to confirm whether the changes in epithelial and endothelial layer integrity revealed by the RTCA-DP system may be related to apoptosis.

## Materials and methods

### Cells

Human intestinal epithelial Caco-2 cells (provided by the Nofer Institute of Occupational Medicine, Lodz, Poland) were expanded in medium consisting of EMEM (Eagle’s Minimum Essential Medium; Sigma-Aldrich, M4526), 2 mM l-glutamin, penicillin (100 U/ml) and streptomycin (100 ug/ml) (Sigma-Aldrich, G6784), heat inactivated 10 % fetal bovine albumin (Immuniq, A15–101), 3 % sodium pyruvate (Sigma-Aldrich, S8636) and 8 % HEPES (Sigma-Aldrich, H0887). Primary human aortic endothelial cells (HAEC) (Lonza, CC-2535) were expanded in endothelial basal medium-2 (EGM-2) (Lonza, Clonetics, CC-3162), supplemented with EGM-2 BulletKit containing (Lonza, Clonetics, CC-3156 and 4176). After getting 80–90 % confluence, Caco-2 cells and HAEC were trypsinized with 0.05 % trypsin with 0.02 % EDTA (SAFC Biosciences, 59417C) for 4 min and neutralized by trypsin neutralizing solution (Clonetics, Lonza, CC-5002) for further experiments.

### Cell culture in the real-time cell electric impedance sensing system (RTCA-DP, ×CELLigence)

The RTCA-DP ×CELLigence system (Roche Applied Science, USA), which operates by tracking electrical impedance signals, enables the cell growth status to be monitored in real time on microelectrode-coated plates. The impedance readout is expressed in arbitrary units as “cell index” (CI), reflecting changes in barrier properties, monolayer permeability, cell number, viability and adhesion, and morphology. The “normalized cell index” (nCI) at a certain time point is acquired by dividing the CI value by the value at a reference time point.

Both trypsinized Caco-2 cells and HAEC were separately seeded on E-16 plates at a density of 10,000 cells per well in proper media. While the Caco-2 cells reached their plateau phase on day 5, the HAEC reached their plateau on day 3 after seeding. After reaching the plateau phase, both Caco-2 cells and HAEC were stimulated with 7-ketocholesterol (10 ug/ml) or 25-hydroxycholesterol (10 ug/ml) and CI changes were observed. Optimal concentrations of stimulatory agents used in this study were determined in a pilot experiment. For both Caco-2 and HAEC cells, the cultures were analyzed 10, 24, and 48 h after induction, as the most significant changes could be seen at these time points.

Additionally, when the experiment was finished, a light microscope analysis of the morphological changes of the cell monolayer was performed.

### Cell culture in monolayers

Both trypsinized Caco-2 cells and HAEC were separately seeded on 12-well plates at a density of 25,000 cells per well in proper media. After reaching 80–90 % confluence, both Caco-2 cells and HAEC were stimulated with 7-ketocholesterol (10 ug/ml) or 25-hydroxycholesterol (10 ug/ml) to assess apoptosis in flow cytometry.

### Flow cytometry analysis of apoptosis

After 24 h of stimulation, both Caco-2 cells and HAEC were trypsinized and apoptosis was assessed in a Beckman-Coulter FC500 flow cytometer (Wladyslaw Bieganski Memorial Hospital Laboratory, Lodz, Poland) using Annexin V-FITC and propidium iodide (PI) co-staining (FITC annexin V Apoptosis detection kit; BD Pharmingen, 556547). Annexin-V (−) and PI (−) cells were considered as living cells, Annexin-V (+) and PI (−) as early-apoptotic cells, Annexin-V (+) and PI (+) as late-apoptotic cells, and Annexin-V (−) and PI (+) as necrotic cells.

### Statistical analysis

The results are presented as mean ± SEM for variables with a normal distribution of values. The distribution of particular variables was verified by the Shapiro–Wilk *W *test, whereas the Levene test was performed to test homogeneity of variances. If the results demonstrated normal distribution and homogenous variance, the significance of differences between two groups was estimated using the Student’s *t* test for independent trials. However, if any of these criteria were not fulfilled, a Mann–Whitney *U* test was used for analysis of the differences between the two groups. All statistical evaluations were performed using Statistica software (StatSoft, Tulusa, OK, USA).

## Results

### The effect of 25-hydroxycholesterol and 7-ketocholesterol on the barrier properties of human primary aortic endothelial monolayer cells (HAEC), as measured by the RTCA-DP

**Fig. 1:**
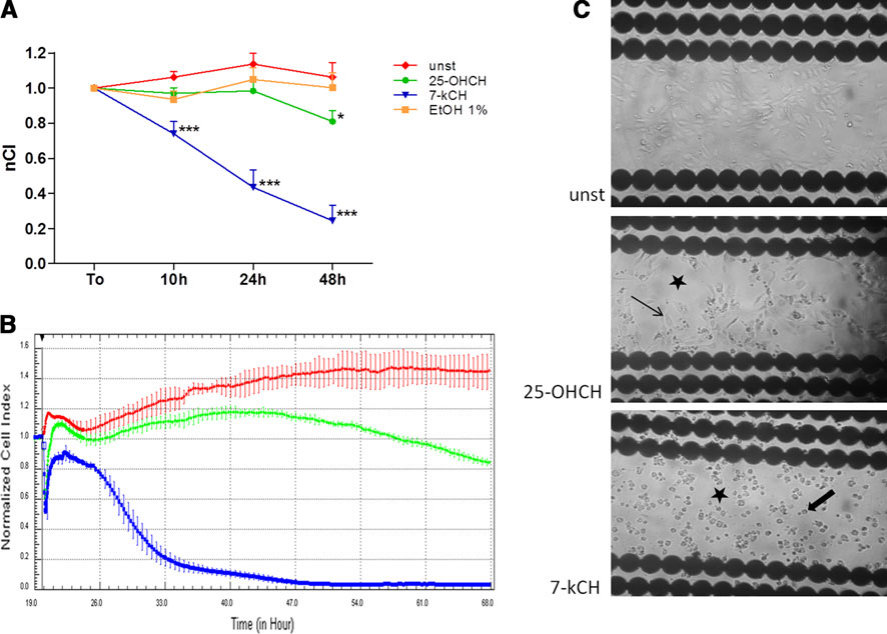
Comparison of the influences of 7-ketocholesterol (10 ug/ml) and 25-hydroxycholesterol (10 ug/ml) on human aortic endothelial cell (HAEC) barrier functions (48 h). **a**, **b** The effect of 7-ketocholesterol and 25-hydroxycholesterol on impedance of HAEC in real-time cell electric impedance sensing system. **a** *n* = 12, from 4 independent experiments, mean ± SEM; *p < 0.05, ***p < 0.001, *nCI* normalized cell index, *unst* unstimulated cells, *7-kCH* 7-ketocholesterol, *25-OHCH* 25-hydroxycholesterol, *EtOH 1* % ethanol 1 %. **b** Representative plot from real-time cell electric impedance sensing system, each line represents the mean of 3 wells of each condition, *red line* unstimulated cells, *green line* 25-hydroxycholesterol-induced cells, *blue line* 7-ketocholesterol-induced cells. **c** HAEC monolayer morphology in E-16 view plate from RTCA-DP: unstimulated, 25-hydroxycholesterol-induced, and 7-ketocholesterol-induced HAEC; *black stars* the damage of endothelial monolayer barrier, *thin arrow* healthy-looking adhering endothelial cells, *thick arrow* round-shaped non adhering endothelial cells (light microscope, ×100)

A RTCA-DP was used to monitor dynamic changes in the barrier properties of HAEC evoked by oxidized cholesterols (Fig. 1a, b). After 10 h of culture, the normalized CI (nCI) value in unstimulated HAEC reached 1.06 ± 0.03, in 25-hydroxycholesterol-induced HAEC it reached 0.96 ± 0.03 (*p* > 0.05), whereas in 7-ketocholesterol-induced HAEC, it dropped significantly to 0.73 ± 0.07 (*p* < 0.001). After 24 h of culture, the nCI value in unstimulated HAEC reached 1.13 ± 0.06, in 25-hydroxycholesterol-induced HAEC it reached 0.98 ± 0.06 (*p* > 0.05), whereas in 7-ketocholesterol-induced HAEC, it dropped significantly to 0.43 ± 0.09 (*p* < 0.001). After 48 h of culture, the nCI value in unstimulated HEAC reached 1.06 ± 0.08, 25-hydroxycholesterol did significantly decrease nCI to 0.8 ± 0.06 (*p* < 0.05), whereas the effect of 7-ketocholesterol on nCI was the strongest, as it dropped significantly to 0.24 ± 0.08 (*p* < 0.001).

At all time-points measured, 1 % ethanol, which was used to dissolve oxycholesterols, did not change the nCI value. As Fig. 1b shows, ethanol affected nCI immediately upon induction, thus destabilizing the monolayer barrier properties of the HAEC. However, this effect lasted for several hours until nCI stabilized, and it did not influence the final effect of the oxysterols.

### Morphological changes induced by 25-hydroxycholesterol and 7-ketocholesterol in the human primary aortic endothelial (HAEC) monolayer

After 48 h of HAEC monitoring in the RTCA-DP system, the morphology of the monolayer seeded on E-16 plates was analyzed by light microscopy (Fig. 1c). In the 25-hydroxycholesterol-induced HAEC, cells with loss of typical endothelial shape were observed, as well as fields of partially destroyed monolayer (black star) next to healthy-looking endothelium (thin arrow). However, in 7-ketocholesterol alone, none of the cells were adherent: all were round-shaped (thick arrow) and floating in growth medium, and complete destruction of the endothelial monolayer (black star) was observed. The 1 % ethanol did not affect the morphology of either the endothelial cells or the monolayer (data not shown).

### The effect of 7-ketocholesterol and 25-hydroxycholesterol on viability and apoptosis of the human primary aortic endothelial (HAEC) monolayer

**Fig. 2:**
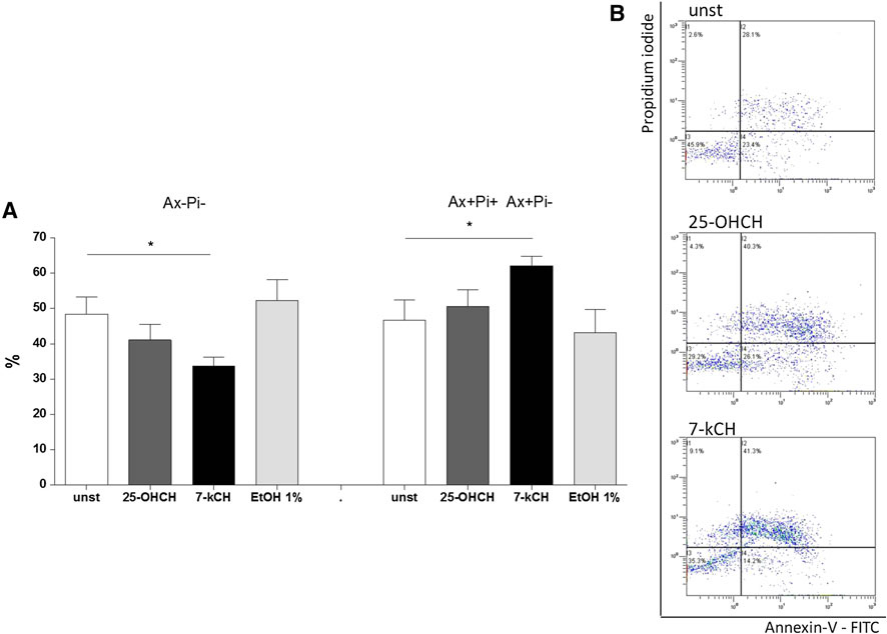
Comparison of the influence of 7-ketocholesterol (10 ug/ml) and 25-hydroxycholesterol (10 ug/ml) on human aortic endothelial cells (HAEC) apoptosis (24 h) assessed by the flow cytometry. **a** *Ax−Pi−* Annexin-V-negative, propidium iodide-negative cells; *Ax+Pi− Ax+Pi+* Annexin-V-positive, propidium iodide-negative cells plus Annexin-V-positive, propidium iodide-positive cells; *n* = 5, from 5 independent experiments, mean ± SEM; *p < 0.05. **b** Representative dot blots of unstimulated (*unst*), 25-hydroxycholesterol-induced (*25-OHCH*) and 7-ketocholesterol-induced (*7-kCH*) Caco-2 cells; *x* axis Annexin V-FITC, *y* axis propidium iodide, flow cytometry

The viability and apoptosis of HAEC were assessed after 24 h of culture in flow cytometry. 25-hydroxycholesterol changed neither the percentage of viable HAEC (Ax– PI−) as compared to unstimulated cells (41.2 ± 4.2 vs. 48.4 ± 4.9 %; *p* > 0.05), nor Ax+ Pi− or Ax+ Pi+ apoptotic cells (50.5 ± 4.7 vs. 46.6 ± 5.8 %; *p* > 0.05) (Fig. 2a, b). On the contrary, 7-ketocholesterol significantly decreased the percentage of viable cells (33.8 ± 2.5 vs. 48.4 ± 4.9 %; *p* < 0.05) and increased the percentage of apoptotic cells (62.1 ± 2.6 vs. 46.6 ± 5.8 %; *p* < 0.05). None of the observed changes in Ax− PI+ cells were statistically significant. Neither HAEC viability nor apoptosis was significantly affected by 1 % ethanol.

### The effect of 25-hydroxycholesterol and 7-ketocholesterol on human intestinal Caco-2 epithelial monolayer barrier properties in real-time cell electric impedance sensing system

**Fig. 3:**
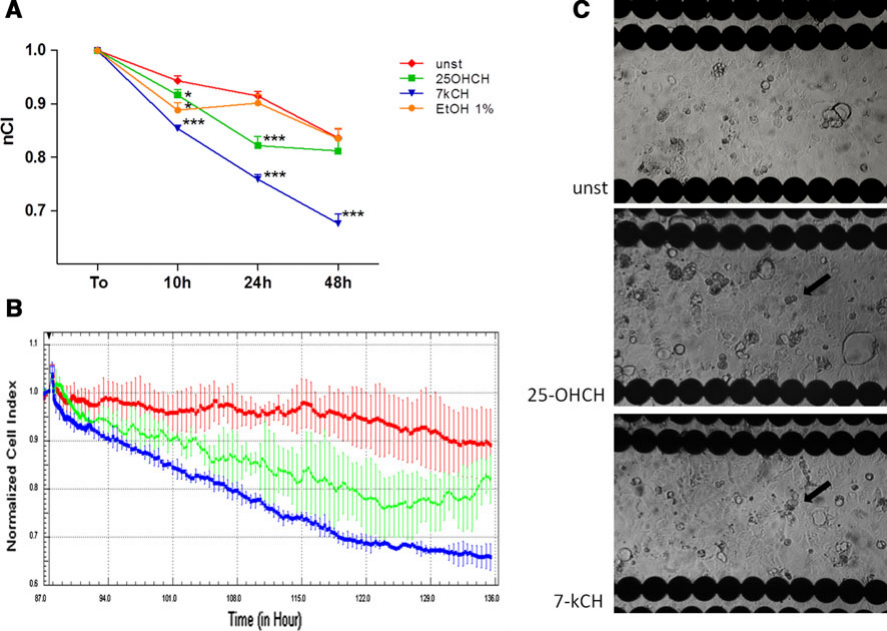
Comparison of the influence of 7-ketocholesterol (10 ug/ml) and 25-hydroxycholesterol (10 ug/ml) on intestinal epithelial Caco-2 cell barrier functions (48 h). **a**, **b** The effect of 7-ketocholesterol and 25-hydroxycholesterol on impedance of intestinal epithelial Caco-2 cells in real-time cell electric impedance sensing system. **a** *n* = 12, from 4 independent experiments, mean ± SEM; *p < 0.05, ***p < 0.001, *nCI* normalized cell index, *unst* unstimulated cells, *7-kCH* 7-ketocholesterol, *25-OHCH* 25-hydroxycholesterol, *EtOH 1* % ethanol 1 %. **b** Representative plot from real-time cell electric impedance sensing system, each line represents the mean of 3 wells of each condition, *red line* unstimulated cells, *green line* 25-hydroxycholesterol-induced cells, *blue line* 7-ketocholesterol-induced cells. **c** Caco-2 cell monolayer morphology in E-16 view plate from RTCA-DP: unstimulated, 25-hydroxycholesterol-induced and 7-ketocholesterol-induced HAEC, *thick arrow* round-shaped non-adhering epithelial cells; (light microscope, ×100)

A RTCA-DP system was used to monitor dynamic changes in the barrier properties of human intestinal epithelial cells (Caco-2) evoked by oxidized cholesterols (Fig. 3a, b). After 10 h of culture, nCI value in unstimulated Caco-2 cells was 0.94 ± 0.01, in 25-hydroxycholesterol-induced Caco-2 cells, it reached 0.91 ± 0.01, and this difference was statistically significant (*p* < 0.05). In 7-ketocholesterol-induced Caco-2 cells, nCI dropped significantly to 0.85 ± 0.005 (*p* < 0.001). The effect of 7-ketocholesterol was significantly greater than 25-hydroxycholesterol (*p* < 0.001). After 24 h of culture, the nCI value in unstimulated Caco-2 cells reached 0.91 ± 0.008. In 25-hydroxycholesterol-induced Caco-2 cells, it reached 0.82 ± 0.01 and it was significantly lower than unstimulated cells (*p* < 0.001), whereas in 7-ketocholesterol-induced Caco-2 cells, nCI dropped significantly to 0.76 ± 0.008 (*p* < 0.001). The effect of 7-ketocholesterol was significantly greater than 25-hydroxycholesterol (*p* < 0.001). After 48 h of culture, the nCI value in unstimulated Caco-2 cells reached 0.83 ± 0.01. In contrast to the previous time-points, 25-hydroxycholesterol did not affect nCI as compared to unstimulated cells (nCI 0.81 ± 0.02; *p* > 0.05), whereas the effect of 7-ketocholesterol on nCI was the strongest, as it dropped significantly to 0.67 ± 0.01 (*p* < 0.001).

As Fig. 3a shows, ethanol affected nCI right upon induction, thus destabilizing Caco-2 cell monolayer barrier properties, and this effect remained in the 10th hour of induction as compared to unstimulated control (0.88 ± 0.01 vs. 0.94 ± 0.01; *p* < 0.05). However, after several hours, nCI stabilized and ethanol did not influence the final effect of 7-ketocholesterol nor in the 24th and 48th hour of stimulation.

### Morphological changes induced by 25-hydroxycholesterol and 7-ketocholesterol in human intestinal Caco-2 epithelial monolayer

After 48 h monitoring of the Caco-2 cells in ×CELLigence, the morphology of the monolayer seeded on the E-16 plates was analyzed using a light microscope (Fig. 3c). In 25-hydroxycholesterol-induced Caco-2 cells, just several non-adherent cells could be found, having lost the typical endothelial shape (thick arrows) were seen as compared to the unstimulated monolayer. However, the field of vision was still covered by normal-shaped adjacent Caco-2 cells, which indicates that monolayer had not been destroyed. The Caco-2 cell monolayer induced with 7-ketocholesterol was observed to have similar morphology. The morphologies of both endothelial cells and monolayer were not affected by 1 % ethanol (data not shown).

### The effect of 7-ketocholesterol and 25-hydroxycholesterol on human intestinal epithelial (Caco-2) cell viability and apoptosis

**Fig. 4:**
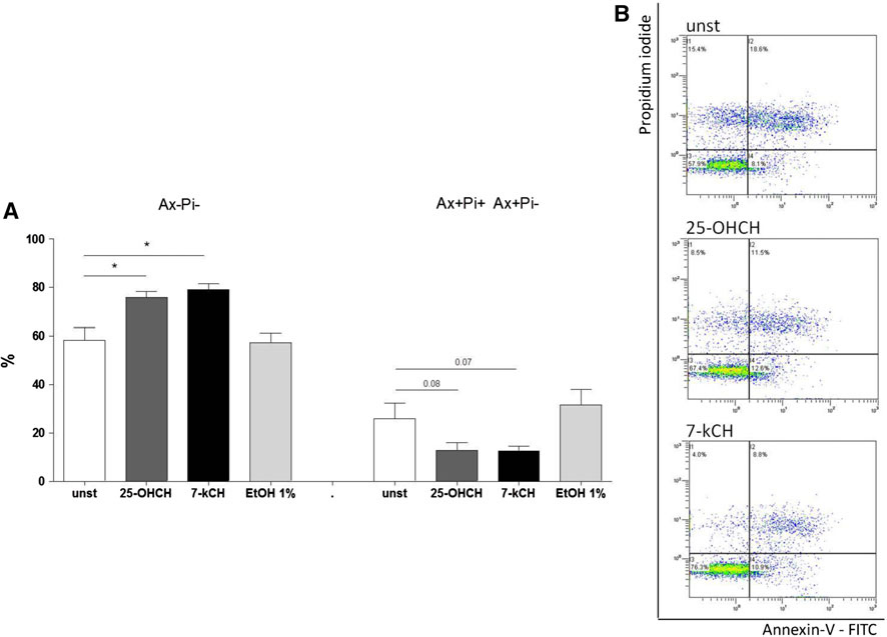
Comparison of 7-ketocholesterol (10 ug/ml) and 25-hydroxycholesterol (10 ug/ml) influence on intestinal epithelial Caco-2 cell apoptosis (24 h) assessed by flow cytometry. **a** *Ax– Pi−* Annexin-V-negative propidium iodide-negative cells;* Ax+Pi− Ax+Pi+ *Annexin-V-positive, propidium iodide-negative cells plus Annexin-V-positive, propidium iodide-positive cells; *n* = 5, from 5 independent experiments, mean ± SEM; *p < 0.05). **b** Representative dot blots of unstimulated (*unst*), 25-hydroxycholesterol-induced (*25-OHCH*) and 7-ketocholesterol-induced (*7-kCH*) Caco-2 cells; *x* axis Annexin V-FITC, *y* axis Propidium iodide, flow cytometry

The viability and apoptosis of the Caco-2 cells were assessed after 24 h of culture in flow cytometry. 25-hydroxycholesterol increased the percentage of viable Caco-2 cells (Ax – Pi ) as compared to unstimulated cells (75.7 ± 2.5 vs. 58.3 ± 5.3 %; *p* < 0.05) and decreased the percentage of apoptotic cells (Ax+ Pi− plus Ax+ Pi+ cells): 12.9 ± 3.1 vs. 25.9 ± 6.4 %; *p* = 0.08 (Fig. 4a, b). Similarly, 7-ketocholesterol significantly increased the percentage of viable cells (79.0 ± 2.5 vs. 58.3 ± 5.3 %; *p* < 0.05) and decreased the percentage of apoptotic cells (12.6 ± 2.0 vs. 25.9 ± 6.4 %; *p* = 0.07). Although the effect of 7-ketocholesterol and 25-hydroxycholesterol on the proportion of apoptotic cells was not statistically significant, they significantly decreased the percentage of all dead cells (combined Ax+ Pi− plus Ax+ Pi+ plus Ax− Pi+ cells), indicating that these agents have a death-preventive effect. None of the observed changes in Ax− PI+ cells were statistically significant. Caco-2 cell viability and apoptosis was not significantly affected by 1 % ethanol.

The comparison of 7-ketocholesterol and 25-hydroxycholesterol on HAEC and Caco-2 cells is summarized in Table 1.

**Table 1:**
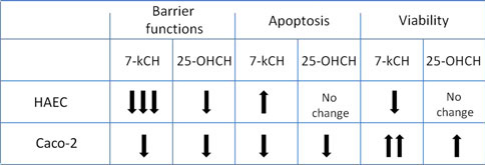
Comparison of the influence of 7-ketocholesterol (10 ug/ml) and 25-hydroxycholesterol (10 ug/ml) on human aortic endothelial cells (HAEC) and intestinal epithelial Caco-2 cell barrier functions and apoptosis: summary

## Discussion

This study is the first to show the effect of oxidized cholesterols on the barrier properties of HAEC and human intestinal Caco-2 epithelium assessed using a RTCA-DP. Our study indicates that both 7-ketocholesterol and 25-hydroxycholesterol decrease the integrity of the endothelial monolayer. However, 7-ketocholesterol was found to have a quicker effect, as although nCI was already significantly lowered after 10, 48 h of stimulation were needed before 25-hydroxycholesterol was observed to have an effect. Secondly, 7-ketocholesterol induced a much greater decrease of monolayer impedance at all analyzed time-points than 25-hydroxycholesterol. The degree of endothelial barrier damage depended on the time of influence—the longer the oxysterols affected the monolayer, the more profound was the observed effect.

The decrease of impedance in the HAEC monolayer induced with 7-ketocholesterol was accompanied by complete destruction of endothelium: it completely lost its confluence, and the cells lost their typical morphology. Firstly, the cells became round-shaped, with some of them passively floating in the growth medium. Secondly, they lost the ability to adhere to the well bottom, suggesting their death. The 25-hydroxycholesterol also reduced the confluence of the endothelial monolayer, although only partially, as fields of untouched endothelium remained next to affected cells, thus indicating its lesser toxicity. Our results are consistent with previous data showing that both 7-ketocholesterol and 25-hydroxycholesterol reduced cell adhesion of bovine aortic endothelial cells [9].

In the next step, the destruction of the endothelial monolayer was confirmed as being due to the increased apoptosis of HAEC: after 24 h, 7-ketocholesterol decreased the percentage of viable cells and increased the percentage of apoptotic cells, which was consistent with the decrease of monolayer impedance. Interestingly, 25-hydroxycholesterol did not significantly affect the viability and apoptosis of endothelial cells. However, it should be remembered that the flow cytometric analysis of apoptosis was performed in the 24th hour of cell stimulation, which is also consistent with measurements in RTCA-DP, and 25-hydroxycholesterol also did not influence monolayer impedance at this time. It cannot be excluded that 25-hydroxycholesterol may have triggered cell apoptosis if this analysis had been extended for longer than 48 h, as similar studies based on the cell type have shown [11]. Nevertheless, the results clearly indicate that 7-ketocholesterol is a much more potent cytotoxic agent than 25-hydroxycholesterol.

Endothelial damage is considered as the earliest step in the initiation of vascular lesions [9]. In 7-oxycholesterol- and oxLDL-treated cells, increased levels of Fas and Fas ligand have been reported; in this way, oxidized cholesterols may contribute to apoptosis [12]. Oxysterols have also been shown to induce apoptotic cell death of human umbilical vein endothelial cells (HUVEC) by early lipid accumulation and lisosomal membrane permeabilization [13]. Our results show that oxidized cholesterols, being disruptive agents, may completely destroy the endothelial barrier within the arterial intima, thus providing oxidized cholesterol, coagulation factors, and a wide range of other blood agents and immune cells with uncontrolled access to media, and enable them to have a direct toxic influence on arterial smooth muscle cells, as well as the migration of immune cells within the vascular wall. It may also be potentially dangerous for smooth muscle cells, as oxysterols have also been shown to induce apoptosis within them [14, 15]. Since damage to the intima is associated with the apoptotic death of endothelial cells, it may also induce a strong inflammatory response. In the case of a particularly cytotoxic oxidized cholesterol, like 7-ketocholesterol, having a permanent effect on the intima, such a rapid cytotoxic effect may not be compensated by increased proliferation of endothelial cells. Therefore, permanent endothelial barrier loss may be a key factor of atherogenesis.

In order to assess whether the loss of barrier properties caused by 7-ketocholesterol and 25-hydroxycholesterol observed in the RTCA-DP is specific to vascular cells, a number of tests were performed on intestinal epithelial Caco-2 cells. The intestinal epithelium forms a barrier separating two compartments in the alimentary system, and, in doing so, it is exposed to wide range of oxidized cholesterols present in foodstuffs, such as dairy products, milk, eggs, and meat [5]. Our study evidenced that both 7-ketocholesterol and 25-hydroxycholesterol decrease the integrity of the epithelial monolayer; however, with particular distinctions. Firstly, the decrease of impedance of Caco-2 monolayer was much less profound than in endothelium throughout the whole time of induction. Secondly, in contrast to the endothelium, 25-hydroxycholesterol was observed to have an effect as soon as after 24 h of incubation; however, surprisingly, it was diminished at 48 h. Similarly, 7-ketocholesterol was found to have a stronger effect than 25-hydroxycholesterol, and the loss of barrier properties of Caco-2 cells was time-dependent.

The decrease of Caco-2 monolayer impedance evoked by oxidized cholesterols was not accompanied by morphological changes observed in the endothelium. Neither 7-ketocholesterol nor 25-hydroxycholesterol affected cellular confluence of the monolayer, typical cell shape, and adherence. Moreover, the slight decreases of impedance caused by both 7-ketocholesterol and 25-hydroxycholesterol were not associated with decreased cell viability and increased apoptosis. Surprisingly, it was accompanied by an enhanced percentage of viable cells and reduced percentage of dead cells, at least after 24 h of stimulation.

Therefore, the slight loss of Caco-2 cell monolayer integrity revealed by RTCA-DP might have been the result of changes in connections between neighboring cells affecting paracellular permeability. As the intestinal epithelium behaves as a permeable filter dynamically regulated by a counterbalance of barrier-protective and barrier-disruptive molecules, loss of its integrity caused by the oxidized cholesterol found in food products may expose dendritic cells patrolling subepithelial space to a wider range of antigens and agents residing in the intestinal lumen. However, this does not lead to uncontrolled access of alimentary lumen agents to deeper layers of intestinal wall.

Bearing in mind that these phenomena were observed in a Caco-2 intestinal adenocarcinoma cell line, one should interpret them with caution, as they may be less prone to apoptotic cell death and may become more resistant when exposed to proapototic stimuli.

They may also use cholesterol for faster proliferation and development. Furthermore, 7β-hydroxycholesterol was shown to induce Caco-2 cell apoptosis and inflammatory response [16]. However, the proapoptotic effect of oxysterols on Caco-2 cells is not so clear, as a mixture of oxysterol showed a slight pro-apoptotic effect on human colon cancer Caco-2 cells [17]. Secondly, susceptibility to the proapoptotic effect of oxysterols may depend on their differentiation. According to Biasi, only differentiated colonic cells were susceptible to the proapoptotic action of the oxysterol mixture [18].

To sum up: 7-ketocholesterol completely destroys the barrier made by a human primary aortic endothelial monolayer, evoking cell apoptosis. This effect is stronger than that of 25-hydroxycholesterol, indicating that the site of cholesterol oxidation might play a pivotal role in its cytotoxicity. An intestinal epithelial Caco-2 monolayer is much more resistant to oxidized cholesterols as it only slightly affect its integrity and increased cell viability. Human primary aortic endothelium is much more vulnerable to the oxidized cholesterols than human intestinal Caco-2 epithelium. Damage to the endothelium by oxidized cholesterol may disrupt local homeostasis and allow open access to inner parts of the vascular wall for lipids, as well as other peripheral blood-derived agents and immune cells, leading to atherogenesis.
